# Inflammation as a driver of hematological malignancies

**DOI:** 10.3389/fonc.2024.1347402

**Published:** 2024-03-20

**Authors:** Sumedha Saluja, Ishu Bansal, Ruchi Bhardwaj, Mohammad Sabique Beg, Jayanth Kumar Palanichamy

**Affiliations:** Department of Biochemistry, All India Institute of Medical Sciences, New Delhi, India

**Keywords:** inflammation, hematopoiesis, hematopoietic stem cells (HSCS), cytokines, RNA binding proteins, leukemia

## Abstract

Hematopoiesis is a tightly regulated process that produces all adult blood cells and immune cells from multipotent hematopoietic stem cells (HSCs). HSCs usually remain quiescent, and in the presence of external stimuli like infection or inflammation, they undergo division and differentiation as a compensatory mechanism. Normal hematopoiesis is impacted by systemic inflammation, which causes HSCs to transition from quiescence to emergency myelopoiesis. At the molecular level, inflammatory cytokine signaling molecules such as tumor necrosis factor (TNF), interferons, interleukins, and toll-like receptors can all cause HSCs to multiply directly. These cytokines actively encourage HSC activation, proliferation, and differentiation during inflammation, which results in the generation and activation of immune cells required to combat acute injury. The bone marrow niche provides numerous soluble and stromal cell signals, which are essential for maintaining normal homeostasis and output of the bone marrow cells. Inflammatory signals also impact this bone marrow microenvironment called the HSC niche to regulate the inflammatory-induced hematopoiesis. Continuous pro-inflammatory cytokine and chemokine activation can have detrimental effects on the hematopoietic system, which can lead to cancer development, HSC depletion, and bone marrow failure. Reactive oxygen species (ROS), which damage DNA and ultimately lead to the transformation of HSCs into cancerous cells, are produced due to chronic inflammation. The biological elements of the HSC niche produce pro-inflammatory cytokines that cause clonal growth and the development of leukemic stem cells (LSCs) in hematological malignancies. The processes underlying how inflammation affects hematological malignancies are still not fully understood. In this review, we emphasize the effects of inflammation on normal hematopoiesis, the part it plays in the development and progression of hematological malignancies, and potential therapeutic applications for targeting these pathways for therapy in hematological malignancies.

## Introduction

Inflammation is one of the defense mechanisms of the body that is utilized to fight against infections and regenerate injured tissues (1). Sustained inflammatory stimuli for an extended period can lead to chronic inflammation. Chronic inflammation can promote the occurrence and development of cancer by promoting blood vessel growth, cancer cell proliferation, and tumor invasiveness (2). Inflammatory biomarkers have been linked to increased cancer risk and mortality (3), and chronic systemic low-grade inflammation is a risk factor for incident cancer (4). Inflammatory biomarkers such as the neutrophil-to-lymphocyte ratio (NLR), systemic inflammation response index (SIRI) and systemic immune-inflammation index (SII) have been found to be predictive of survival for multiple types of cancers (5). Hematopoiesis is a highly controlled process maintained by the division of quiescent, self-renewing, multipotent hematopoietic stem cells (HSCs) and lineage-specific downstream progenitors in the bone marrow (BM). Cell extrinsic signals like chemokines and cytokines activate HSCs and cells in the microenvironment that help compensate for normal cellular loss (6). Inflammation is known to play a significant role in normal hematopoiesis.

A majority of hematological malignancies result from mutations in the hematopoietic stem cells, leading to uncontrolled growth and proliferation of HSCs. While inflammation is known to play a beneficial role in immune system activation and tissue regeneration, chronic inflammation can lead to HSC damage, resulting in bone marrow failure or the development of leukemia (7).

**Figure 1:**
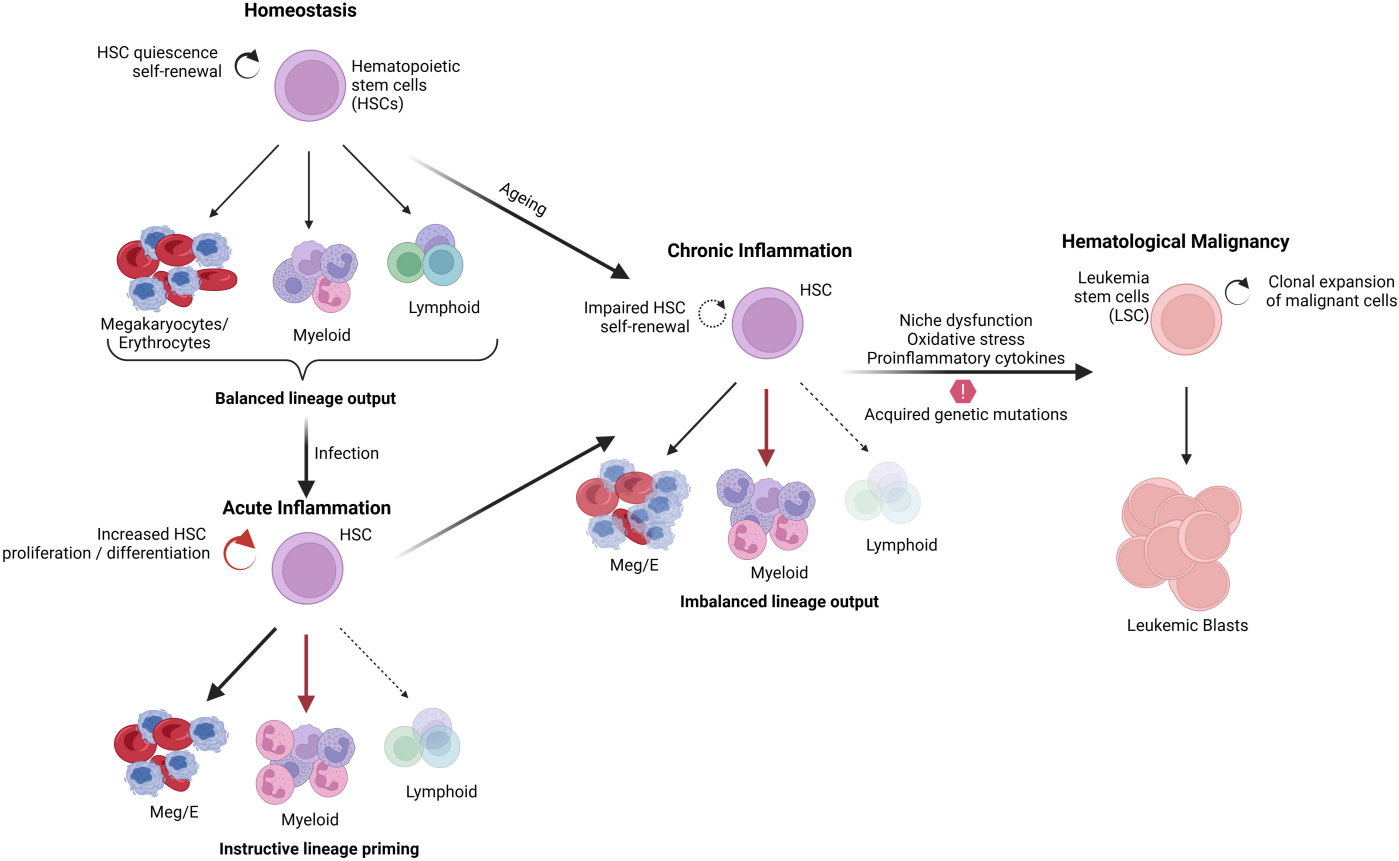
Effect of inflammation in normal hematopoiesis and malignant transformation.

Usually, stress or infection causes the activation of numerous signaling pathways, which increases the production of inflammatory cytokines and chemokines, which activate immune cells like B and T lymphocytes, helping to eradicate the infection. Inflammation regulates homeostasis and maintains the hematopoietic system. However, uncontrolled inflammation can also play pathogenic roles by disrupting homeostasis, which can potentially contribute to tumor development. A variety of cells of the immune response release pro-inflammatory cytokines, including IL-1β, IL-1α, TNFα, IL-6, IL-12, IFN-γ, and chemokines such as CCL2 and CXCL12, which participate in the initiation, growth, and progression of tumor and development of drug resistance (8, 9). Transcription factors involved in inflammation, such as NFκB and STAT3, are known to promote the development and progression of cancer by controlling the expression of genes involved in apoptosis, cell proliferation regulation of angiogenesis, tumor metastasis and invasion (8, 10, 11). Thus, the inflammatory elements, including cytokines, chemokines, and their receptors, play a significant role in tumorigenesis and promote the survival of tumor cells with metastatic and invasive properties (9, 12). In addition, chronic inflammation often leads to the overproduction of hematopoietic stem cells, which subsequently undergo DNA mutations, thereby leading to the development of hematological malignancies (13) (**Figure 1**).

The recent advances in molecular biology and the development of genetically modified mouse models helped unravel the various aspects of inflammation during tumorigenesis (14, 15). Therefore, it is crucial to understand the molecular mechanisms that connect inflammatory processes to tumorigenesis and metastasis, which can be targeted for therapeutic and diagnostic purposes. This review highlights the role of inflammation in normal hematopoiesis and the relevance and requirement of the inflammation process to be tightly controlled at both initiation and termination, which when lost, can lead to the development and progression of cancer.

## Effect of inflammation in normal hematopoiesis

Hematopoiesis is the formation of all blood cells and components of blood plasma hierarchically controlled by HSCs. The multipotent HSCs can give rise to all types of blood cells residing in the bone marrow (16). HSCs possess self-renewal ability, which helps maintain the stem cell pool. Although quiescent, HSCs undergo division in response to external stimuli like infections and irradiation (17). Stress conditions like inflammation can cause HSCs to undergo proliferation. During inflammation, HSCs express receptors for cytokines and chemokines that help HSCs recognize signals from immune cells to adapt their cycling and differentiation potential (18, 19). However, mutations in HSCs can lead to uncontrolled proliferation, which can cause bone marrow damage or result in hematological malignancies (17). These mutations primarily affect the self-renewal ability of HSCs, activating their proliferation and inhibiting the production of mature cells.

Somatic mutations in genes such as *NPM1*, which is involved in the maintenance of HSCs’ quiescence and self-renewal, and *TET2*, *DNMT3A*, involved in mediating HSCs’ differentiation, results in the transformation of HSCs thereby leading to the development of Acute Myeloid Leukemia (AML) (20). Mixed lineage leukemia (MLL) is known to be commonly rearranged in leukemia. Knockout of the MLL gene led to a reduction in HSC numbers in mice, and HSCs deficient in the MLL gene are unable to reconstitute the bone marrow of the recipient mice, signifying that the MLL gene is important for the maintenance of self-renewal ability of HSCs. Since the JAK-STAT signaling pathway activates HSCs to proliferate in response to the release of inflammatory cytokines, gain of function mutations in JAK2 non-receptor tyrosine kinase can often result in long-term activation of HSCs, sustained differentiation to the erythroid and myeloid lineages and results in AML development (21).

## The HSC niche and inflammation

HSCs reside in the bone marrow within a specific microenvironment termed the HSC niche. The HSC niche comprises the extracellular matrix along with distinct cell types like mesenchymal stem cells (MSCs), osteoblasts, osteoclasts, osteolineage progenitor cells, endothelial cells (ECs) and specialized CXCL12-abundant reticular (CAR) cells and leptin receptor (LEPR) positive cells (22–28). HSCs reside in the perivascular region of sinusoids and arterioles near MSCs and ECs which are essential for maintenance of quiescence and differentiation of HSCs (29). MSCs regulate HSCs via the expression of CXCL12, angiopoietin-1 and vascular cell adhesion molecule-1 (VCAM-1) (30–33). HSCs exist in two distinct niches - endosteal and vascular niche. The endosteal niche, which contains osteoblasts, regulates the quiescence associated with HSCs and their migration to the vascular niche (34). Inside the vascular niche, that contains endothelial cells, HSCs undergo differentiation (27, 28, 35, 36). TGF-β is secreted by non-myelinating Schwann cells around the blood vessels in the bone marrow (37). Additionally, megakaryocytes around sinusoids are responsible for maintaining HSCs quiescence, through release of factors such as TGF-β along with Platelet factor (PF-4/CXCL4) and thrombopoietin (38–41). Studies with conditional deletion of CXCL12 in endosteal and vascular niche of HSCs have highlighted role of CXCL12 in the maintenance and self-renewal of HSCs. This is controlled by CXCL12 released by immature mesenchymal stem and progenitor cells, with a smaller contribution from endothelial cells (42, 43).

Hematopoietic stress conditions like systemic inflammation or infections cause HSCs to exit their quiescence and undergo proliferation and differentiation to compensate for cellular loss. The production of mature myeloid cells, including neutrophils and monocytes from HSCs, as a result of inflammation, is termed emergency myelopoiesis (25, 26, 44). The HSC niche plays a significant role in mediating the hematopoietic response to peripheral or systemic inflammation. The HSC niche secretes certain factors such as granulocyte colony-stimulating factor (G-CSF) that promote myelopoiesis during inflammation (45–47). G-CSF is the central regulator of inflammation induced emergency myelopoiesis. It is the endothelial cells which have been characterized as the main source of production of G-CSF during inflammation. During LPS-induced systemic inflammation, TLR4 signaling in endothelial cells led to elevated G-CSF synthesis, resulting in emergency granulopoiesis (7, 19, 45–47).

Inflammation induces the expression of G-CSF in ECs and IL-6 from ECs and MSCs (48). CXCL12 and Kit ligand (KITL) are required to maintain HSCs in the BM niche (27, 47). Upon inflammation, the expression of CXCL12 and KITL is downregulated (42). In order to mediate HSCs mobilization in the niche, G-CSF acts on different cell types such as MSCs, macrophages, neutrophils and osteolineage cells (49). G-CSF affects osteoblastic activity and inhibits the expression of CXCL12 directly or through functional changes in macrophages or granulocytes (48, 50). In the case of viral infections, Interferon-γ (IFN-γ) secreted by CD8^+^ T cells acts on MSCs in the HSC niche and leads to increased release of IL-6 by MSCs (51). In summary, HSCs undergo transient division in response to stress conditions such as inflammation to give rise to myeloid cells to compensate for cellular loss. At the same time, these HSCs further secrete pro-inflammatory cytokines such as IL-6, which will activate themselves in a paracrine or autocrine manner to mediate the expansion of HSCs in the bone marrow (52, 53).

## Role of inflammation in hematological malignancies

Inflammation in the bone marrow has been reported to contribute to the development of hematological malignancies (54). Myeloid malignancies like AML, myeloproliferative neoplasms (MPN), myelodysplastic syndromes (MDS) are thought to represent a clonal disease of HSCs (55). Point mutations and mosaic chromosomal alterations increase the risk of lymphoid malignancies like chronic lymphocytic leukemia (CLL), small lymphocytic lymphoma (SLL), and diffuse large B-cell lymphoma (DLBCL) (56).

These malignant clones are called leukemia stem cells (LSCs) or leukemia initiating cells (LICs). LSCs were initially characterized in AML patients. The surface immunophenotype for LSCs is usually CD34^+^ CD38^–^ CD90^–^ along with interleukin-3 receptor (IL-3R), and CD117 positivity. These LSCs have also been identified in CML and ALL cells that carry the BCR-ABL fusion gene (54). The LSCs share surface phenotypic markers with HSCs during the evolution of leukemia. This further leads to the production of both the clonogenic leukemic progenitors and the non-clonogenic blast cells that eventually leads to full-blown leukemogenesis (57). Like HSCs, LSCs also exhibit self-renewal, quiescence, and multipotency properties but also uncontrolled proliferation (57, 58). This subpopulation of leukemia cells has properties of HSCs and, along with loss of differentiation and apoptosis, leads to cancer development. These stem cell-like features make it challenging to target LSCs and render LSCs resistant to conventional chemotherapy, thereby leading to the relapse of the disease (59).

HSCs, when mutated, can be the source of the generation of LSCs. HSCs usually have a finite lifespan. However, the self-renewing HSCs, when mutated, sustain for a long time, allowing genetic damage and malignant transformation of HSCs to LSCs (60). AML arises from multiple genetic mutations that lead to increased proliferation, survival, and impaired differentiation of hematopoietic progenitor cells (61). The most recurrent mutations in AML occur in genes such as *FLT3, NPM1, CEBPA, IDH1/IDH2, DNMT3A*, and *RUNX1* (62). For example, internal tandem duplications in the *FLT3* gene (*FLT3-ITD*) and mutations in the nucleophosmin (*NPM1*) gene are detected in approximately 30% and 50% of AML cases, respectively (63). Other common mutations include those in the DNA methyltransferase 3A (*DNMT3A*) gene, present in 20-30% of cases (64). The order in which these mutations are acquired can influence leukemia development. A frequent sequence is an initial DNMT3A mutation, followed by an *NPM1* mutation and then *FLT3-ITD* mutation (60). *DNMT3A* mutations showed higher levels of pre-leukemic stem cells that are resistant to chemotherapy and thus further lead to leukemia development. Injection of *DNMT3A* mutant pre-leukemic HSCs in NSG mice demonstrated a competitive repopulation advantage over non-mutated HSCs (60). Like HSCs, the maintenance of LSCs also relies on their tumor microenvironment, termed the LSC niche. The chemokine CXCL12 binds to its receptor, CXCR4 and plays a prominent role in the homing of HSCs and LSCs in the bone marrow by mediating adherence of AML cells to stromal cells, leading to proliferation and resistance from chemotherapeutic drugs. It has been known that CXCR4 is highly expressed in AML and ALL patients and results in poor prognosis (65–67). Transforming growth factor β (TGF-β) acts as a critical regulator of quiescent G0 state in the AML and CML cells, thereby maintaining LSCs (68). Besides soluble factors, LSCs also interact with niche cells via cell-cell interactions. For example, CD44, a transmembrane glycoprotein, mediates the adhesion of LSCs to the niche and transduces intracellular signals involved in proliferation and differentiation. CD44 targeting led to the loss of migration of human and murine LSCs to the niche, leading to the eradication of LSCs (68–71).

**Figure 2:**
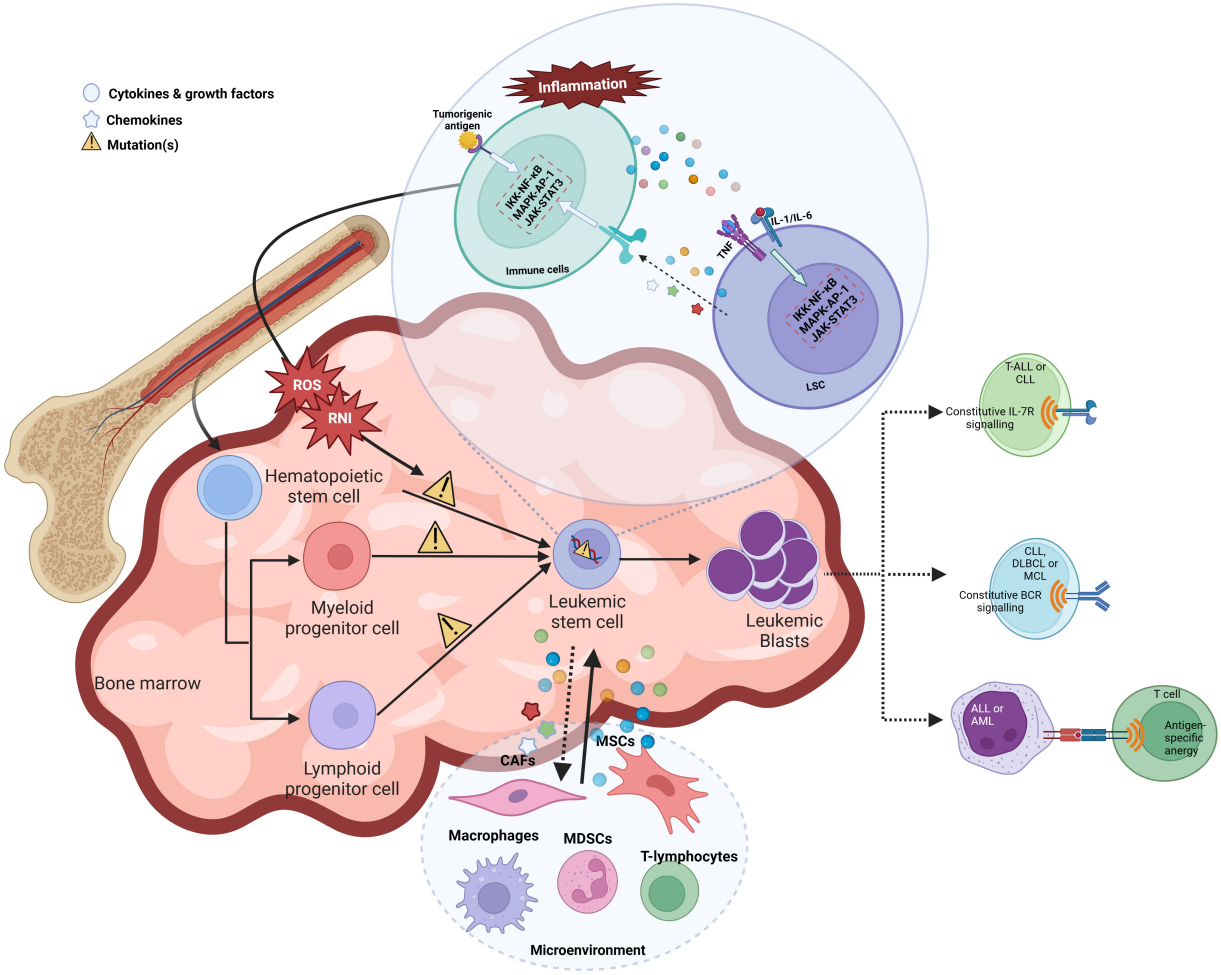
Chronic inflammation leads to leukemogenesis by various mechanisms.

Inflammation can trigger oncogenesis either by cell extrinsic or intrinsic mechanisms. The extrinsic mechanism is driven by external factors, including inflammatory conditions and micro-environmental factors, where a constant inflammatory state contributes to tumor initiation and progression. For instance, patients with inflammatory bowel disease have shown increased susceptibility to lymphomas, leukemias, and hepatocarcinoma. However, the cell-intrinsic pathway involves genetic alterations affecting oncogenes, tumor suppressors, and genome stability genes, which activate inflammatory pathways (such as the NFκB pathway), thereby generating an inflammatory microenvironment in tumors (72) (**Figure 2**).

**Figure 3:**
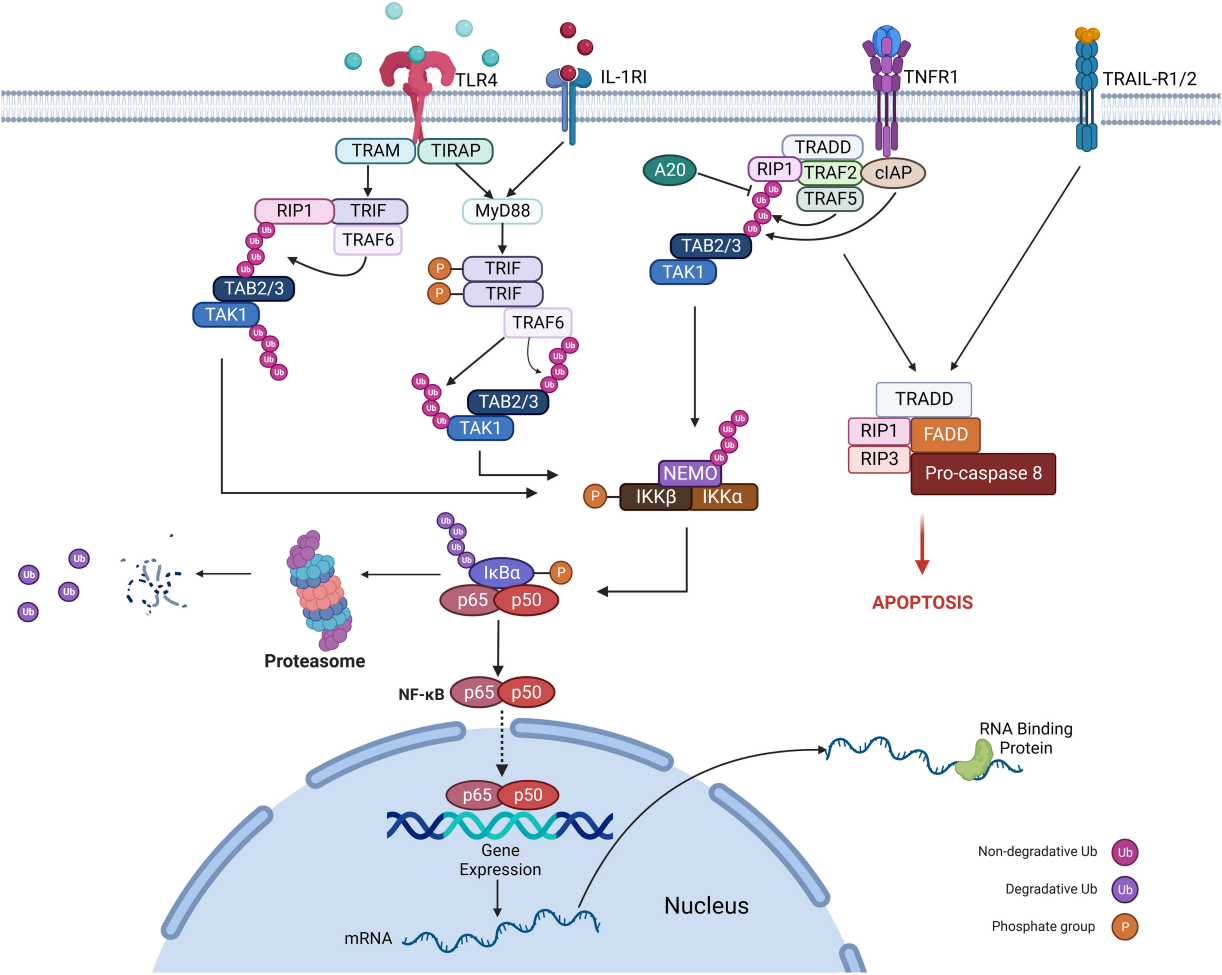
Mechanism of regulation of the NFκB pathway.

In both malignant and inflammatory cells, NFκB is activated downstream to the TLR-MyD88 pathway (sensing microbes or tissue damage) or the inflammatory cytokines, including TNF and IL-1β (**Figure 3**). It has been known that NFκB is one of the primary inflammatory pathways associated with myeloid and lymphoid malignancies (73, 74). Alternatively, NFκB activation can result from genetic alterations (amplification, mutations, or deletions) in cancer cells. NFκB enhances the expression of antiapoptotic genes like *BCL2*, *CLIP*, and *cIAP* and increases the survival of tumor cells (75).

Inflammatory mediators like S100A8/A9 or inflammatory cytokines such as TNF-β, IL-1β, IL-6 and IFN-γ, chemokines like CCL2 and CXCL8 are seen to be upregulated in malignancies like myelodysplastic syndrome (76). These molecules contribute to carcinogenesis via the NFκB pathway and STAT3 signaling pathway. MSCs are a population of stem cells that are important in maintaining bone marrow microenvironment. MSCs also have immunoregulatory functions and maintain the immune BM microenvironment by saving the HSCs from stress stimuli. The dysregulated development of MSCs has been shown to lead to MDS induction and further AML development (77). The NFκB pathway is also upregulated in MSCs of patients with MDS. Along with NFκB, STAT3 has also been associated with cancer-related inflammation. NFκB is activated in two ways: a) IKK activity dependent (pro-inflammatory stimulus-dependent) and b) constitutive activity, which is proinflammatory stimulus-independent (73). STAT3 is a transcriptional factor which maintains constitutive expression of NFκB by acetylation of RelA. STAT3 also has a feedback inhibition on IKK activity. B16 mouse melanoma tumor cells and DU145 prostate cancer cells, showed high STAT3 activity by increasing phosphorylation of RelA protein in the presence of TNF-α. B16 melanoma cells showed a reduction in RelA phosphorylation and, thus, in NFκB activity after STAT3 knockdown (73, 78). Tumor cells have high expression of VEGF, IL-10, and IL-6; these tumor cells also highly express STAT3. High expression of STAT3 is shown to inhibit DC maturation, which in turn leads to immature DC accumulation and subsequent immunosuppression, leading to tumor cell escape. Stat3 deletion in HSCs improved DC maturation and function which induced antitumor activity in mice. Targeting STAT3 in cancer cell lines and *in-vivo* studies has also reduced tumor growth (79, 80).

STAT proteins are also involved in hematopoietic growth factor signal transduction. Signaling molecules activate different STATs: thrombopoietin (TPO) activates STAT3, whereas granulocyte-macrophage (GM)–CSF, TPO, and IL-3 activate STAT5. It is known that STAT1, 3 and 5 have all been overexpressed in acute and chronic leukemia (81).

A study conducted on Tet2-deficient mice showed increased IL-6 production in response to microbial infection, resulting in preleukemic myeloproliferation, signifying the critical role of inflammation in the progression of leukemia (82). A study using the MLL-AF9-induced AML mouse model showed that the leukemia cells expressed factors like TNF and CXCL12. These led to the remodeling of endosteal vessels, generating a niche that supports the overall proliferation of malignant hematopoietic clones- LSCs and a decrease in the number of normal HSCs (83).

Taken together, these studies implied the need to focus on a better understanding of the mechanisms of crosstalk between inflammation and its effect on interactions of HSCs with their niche during normal hematopoiesis and emergency myelopoiesis which may subsequently lead to the development of leukemias (**Table 1**).

**Table 1:** Common targets and inhibitors in clinical trials/approved for targeting hematological and inflammatory disorders.

Targets	Approved inhibitors	Inflammatory disorders	Hematological disorders	References
Bruton tyrosine kinase	Ibrutinib (first generation)	Graft-versus-host disease (GvHD), COVID-19, RS	CLL, MCL, ALL Marginal zone lymphoma (MZL), Phase 3: AML Phase 2: DLBCL, HCL, MM	(84–86)
	Acalabrutinib (second generation)	COVID-19, wAIHA, RS, RA	Approved for: CLL and R/R MCL Phase 3: DLBCL, Phase 1: MZL, MM, and AML	(84–86)
	Zanubrutinib	Immune thrombocytopenic purpura (ITP), Phase 2: NMOSD, RS, COVID-19	Approved for: R/R MCL; WM, and R/R MZL Phase 3: hemophagocytic lymph histiocytosis, CLL, and DLBCL	(86, 87)
PI3K D	CAL-101	Allergic rhinitis	B-cell lymphoma	(88)
c-Kit, PDGFRA, PDGFRB, FLT3, PKC, CDK1, SYK, VEGFR-2	Midostaurin	Cutaneous Mastocytosis	Acute Myeloid Leukemia,	(89)
SF3b Complex	H3B-8800		Acute Myeloid leukemia	(90)
COX-II, VEG-F, HIF1-α, NFκB, Cereblon (CRBN)	Pomalidomide	Asthma	Multiple Myeloma	(91–93)

## Inflammation in myeloid malignancies

Myeloid malignancies result from genetic and epigenetic alterations in the myeloid progenitor cells involved in self-renewal and differentiation. Epigenetic modifications and changes in the microenvironment are known to be significant causes of these diseases. There are different types of myeloid malignancies, namely AML, Chronic Myeloid Leukemia (CML), MPNs, myelodysplastic syndrome (MDS) and chronic myelomonocytic leukemia (CMML).

AML is the most diverse hematological malignancy frequently seen in adults (94). The leukemic blasts produced by abnormal myeloid stem cells accumulate in the bone marrow, peripheral blood and other tissues, which reduces the population of normal blood cells and increases the risk of secondary infections (95, 96). Myeloproliferative neoplasms are caused by the dysfunction of multipotent hematopoietic stem cells and clonal myeloproliferation. In addition, genetic rearrangements like BCR-ABL and ETV6-RUNX1 and mutations in the tyrosine kinases like JAK kinases, Abelson (Abl) kinase, activated cdc42 (ACK) Kinases lead to abnormal proliferation (97, 98). Chronic Myeloid leukemia (CML) is usually associated with the BCR-ABL fusion gene (9;22 translocations), also known as the Philadelphia (Ph) chromosome.

Recent studies showed that alterations in the inactive HSCs and reduced interaction with BM niches could lead to their leukemic transformation and myeloid leukemia development (68, 99).

Many signaling pathways are involved in the regulation and development of normal HSCs. Proteins associated with Wnt signaling pathways help in maintaining the stemness of HSCs by regulating their quiescence and self-renewal properties (100). The regulation of HSCs' proliferation, self-renewal, and differentiation depend on various intermediates like cell cycle regulators, cyclin-dependent kinase inhibitors (CKIs), D-cyclins, p18/INK4, PTEN and many other transcription factors like HoxB4 and HoxA9 (101–103). Extrinsic regulatory pathways like Notch, TGF, Sonic Hedgehog, Smad, and Wnt also regulate HSCs' proliferation and self-renewal (103).

JunB is an essential protein responsible for regulating the proliferation and differentiation of long-term HSCs via TGF-β and Notch-Signaling pathways (101) and plays a vital role in maintaining HSCs. Its inactivation by epigenetic modifications has also been reported with the development of myeloid malignancies (104). The niche osteoblasts in CML secrete IL-1β and TNF-α pro-inflammatory cytokines, further enhancing myeloid cell proliferation and, thus, disease progression (30).

The STAT proteins are shown to be essential for myeloid differentiation. Chronic myeloid leukemia, commonly with BCR-ABL translocation, showed continuous expression of STAT3 and STAT5, leading to enhanced expression of BCL-XL, which is an anti-apoptotic BCL2 family protein (81, 105). Erythropoietin is also known to elicit phosphorylation and activate STAT5, which further helps in HSC differentiation (81, 106, 107). STAT5 inhibition led to reduced proliferation of the leukemic cells by enhancing apoptosis and inducing cell cycle arrest (108).

Tumor-associated macrophages (TAMs) are the resident macrophages in the tumor microenvironment, which promote tumorigenesis and angiogenesis, provide an immunosuppressive environment, and contribute to the poor prognosis of the disease (109). TAMs are functionally compromised. Phagocytosis inhibition happens due to the overexpression of a transmembrane protein, CD47, which interacts with the protein, signals regulatory protein alpha (SIRPα), and leads to inhibition of phagocytosis. This protein is highly expressed in LSCs, and its inhibition by anti-CD47 antibody led to an increase in phagocytosis by macrophages and increased the survival rate of myeloid mouse models (110, 111).

The cytokine TRAIL (tumor necrosis factor a-related apoptosis-inducing ligand) mediates apoptosis by caspase -8 mediated pathway. TRAIL can bind to four distinct receptors : TRAIL-R1 and TRAIL-R2 (also known as DR4 and DR5) are functional receptors which contain cytoplasmic death domains and can transduce cell death signals (112). In contrast, TRAIL-R3 and TRAIL-R4 (DcR1 and DcR2), are the truncated receptors and can block TRAIL induced apoptosis (112). It is also known that the binding of TRAIL with TRAIL-R4 leads to NFκB activation and further inflammation (113, 114). The expression of TRAIL-R1 and TRAIL-R2 is reported to be high in AML patients, and there has been evidence which shows high co-TRAIL-R3 expression linked to poor overall survival of the patients (111). TRAIL-R3 is a decoy protein which can be bypassed by targeting TRAIL-R1 and TRAIL-R2 by antibodies and thus can be used as a treatment for AML patients (112). Recombinant soluble TRAIL (rsTRAIL) has shown induction of apoptosis in cancer cell lines (115) including myeloid-leukemia cell lines (58). In addition, the activator of p53, Nutlin-3, and TRAIL enhances apoptosis in AML primary cells when wild-type p53 is present as it enhances apoptosis (115–117).

NFκB is highly expressed in AML patients and LSCs as compared to HSCs. IKKβ is a catalytic subunit of the IKβ complex, which activates the NFκB pathway. IKKβ deletion in the myeloid lineage using a LysM-Cre mouse model reduced tumor growth as well as proinflammatory cytokines without affecting apoptosis (118). IKKβ has been associated with inflammation and carcinogenesis as IKKβ activates factors like COX-2, MMP-9, MIP-2, and KC in myeloid cells, which are pro-inflammatory and linked to tumor development (118, 119).

The pro-survival protein myeloid cell leukemia (MCL-1) is an anti-apoptotic protein that regulates cell cycle progression and mitochondrial homeostasis. MCL-1 has been reported to be overexpressed in multiple myeloid malignancies like multiple myeloma and acute myeloid leukemia (120). Inflammation can contribute to overexpression of MCL-1 with inflammatory cytokines such as IL-6 and IL-8 enhancing MCL-1 transcription (121). Small molecules like AZD5991 which specifically inhibits MCL-1 showed a significant reduction in tumor growth in an OCI-AML3 mouse xenograft model. Further reduction in tumor growth was observed when the drug was administered with Venetoclax, a Bcl-2 inhibitor. Clinical trials for AZD5991 were also approved (120). Other MCL-1 inhibitors like AMG 176 (Amgen) S64315 (MIK66) are also under clinical trial for AML (120). Indisulam is a sulfonamide, targets several components of the cell cycle. It is known to target the G1 phase of the cell cycle and causes a blockade in the G1/S transition through the inhibition of the activation of both CDK2 and cyclin E (122). A phase 2 trial for Indisulam along with Idarubicin and Cytarabine was conducted which showed improved prognosis and increased survival rate in AML and high-risk MDS patients (123). E7820, another sulfonamide, is in phase II clinical trials for solid cancers (124). It acts as an inhibitor of Integrin α2 (ITGA2), which plays a key role in methotrexate-induced epithelial-mesenchymal transition (EMT) in alveolar epithelial cells (125). E7820 also selectively targets RNA splicing factor RBM39 for proteasomal degradation via DCAF15-E3-ubiquitin ligase. This action of E7820 has been observed to induce rapid loss of RBM39, accumulation of splicing errors, and growth inhibition in a DCAF15-dependent manner (125, 126). Interestingly, DCAF15 is found to be more highly expressed in Acute Myeloid Leukemia (AML) patient samples compared to normal hematopoietic progenitors. Therefore, the effects of E7820 in hematological malignancies (such as AML) are also being investigated (127).Targeting the inflammatory pathways that lead to MCL-1 overexpression may provide an alternative approach to inhibiting this anti-apoptotic protein in myeloid malignancies.

The phase I trials using anti-PD1 or anti-CTLA4 (ipilimumab) drugs as a monotherapy failed in both AML and MDS (128–130). Nivolumab, an immune checkpoint inhibitor in combination with azacitidine, a DNA methyltransferase inhibitor is under phase II trial (NCT02397720) for Refractory/Relapsed (R/R) and newly diagnosed AML patients (131).

Chimeric Antigen Receptor -T (CAR-T) cells against ligands which are expressed only on malignant cells is a new approach towards eliminating cancer. Overexpression of NKG2D ligands is seen in solid as well as hematological malignancies. However, the expression of NKG2D is seen to be absent/low in healthy tissues. CAR-T cells with a single infusion of human NKG2D were used in the phase I trial of AML, MDS and multiple myeloma patients which showed limited expansion and persistence of CAR-T cells (132).

It is known that BCR-ABL kinase leads to upregulation of activation-induced cytidine deaminase (AID) that leads to increased genetic instability. AID expression has been associated with blast crisis progression in CML and increases leukemogenesis in BCR-ABL^+^ B-ALL (133). It has been recently investigated that inflammation contributes enhanced expression AID through NFκB pathway and further increases malignancy in BCR-ABL^+^ B-ALL (134). BCR-ABL tyrosine kinase inhibitors like imatinib mesylate was the first drug approved for CML. Nilotinib (second generation inhibitor) and ponatinib (third generation inhibitor) are effective against BCR-ABL mutations like T315I, Y253H, and F317L (135–137). Omacetaxine is an inhibitor of protein translation which has been approved for CML therapy. It hinders the process of protein translation by blocking the initial elongation phase of protein synthesis. It interacts with the ribosomal A-site and impedes the precise arrangement of the side chains of amino acids in incoming aminoacyl-tRNAs. This drug degrades BCR-ABL proteins by inhibiting heat shock protein 70 in a dose dependent manner in imatinib resistant K562 cells (138). Many clinical trials with a combination of such therapies are being carried out in CML and AML patients (135).

RNA-binding proteins (RBPs) play a pivotal role in co and post transcriptional modifications. These RBPs are responsible for genetic alterations and diseases including cancers. TCGA data shows around 484 RNA-binding proteins which have been associated with myeloid malignancies. Out of these, approximately 50 percent are dysregulated in AML. There have been evidences which show dysregulation of RBPs associated with splicing in AML and hence is a potential therapeutic target. The clinical trials for H3B-8800 which targets SF3b splicing complex is one of the studies supporting development of therapeutic agents for myeloid malignancies (90, 127).

## Role of inflammation in lymphoid malignancies

ALL is a group of malignancies of immature B or T cells that occurs predominantly in children. B-ALL constitutes 80-85%, while T-ALL accounts for 15% of pediatric to 25% of adult ALL cases (139).

### B-ALL

B-cell acute lymphoblastic Leukemia is characterized by uncontrolled production of hematopoietic B-precursor cells. The chromosomal translocations that give rise to fusion proteins with oncogenic function and alteration in the role of B-lymphoid transcription factors such as Ikaros, E2A, EBF1 and PAX5 are known to be the causes of B-ALL. Pre-B-ALL cells have been shown to produce high levels of TNFα and IL-6, representing the inflammatory microenvironment's role in this disorder (140). The pro-inflammatory factors IL-1α, IL-1β, and TNFα were highly overproduced in supernatants derived from mononuclear cells of B-ALL patients when compared to their standard counterparts. Cytokines such as G-CSF, GM-CSF, IFNα, IL-12 and IL-7 were substantially elevated in B-ALL patients mediated by the activation of NFκB and STAT3 pathways (141). CCL2 and IL-8, chemokines that suppress normal hematopoiesis, are increased in the BM microenvironment and tend to promote the capacity of BM stromal cells to support the adhesion of ALL cells, indicating that elevated levels of CCL2 and IL8 could indirectly confer survival advantage to ALL cells (142).

Treatment of primary B-ALL patient samples with TRAIL (Apo2 ligand), an anti-cancer cytokine, showed modest apoptotic activity which was heterogeneous (143). However, TRAIL treatment of pre-B-ALL leukemia xenografts induced apoptosis in LICs and LSCs (144).

TRAIL-R1 monoclonal antibody (Mapatumumab) is in phase-II clinical trials for relapsed or refractory Non-Hodgkin's Lymphoma (NHL) as monotherapy and for multiple myeloma as combination therapy with Bortezomib. Dulanermin is recombinant TRAIL which triggers apoptosis via activation of DR4 and DR5 and is in phase III clinical trial for B-NHL patients who have progressed following rituximab therapy. Circularly permuted TRAIL (CPT) based combination therapy with Thalidomide is in phase III trials for R/R MM (145).

Furthermore, in B-ALL patients, increased peripheral levels of CXCL12 and high expression of CXCR4 on leukemic pre-B cells contribute to their proliferation, survival and homing to the BM microenvironment, which is mediated by STAT5, Rac-1 GTPase and a unique p38MAPK signaling pathway (67, 146–148). In childhood ALL, it is found that overexpression of the chemokine receptor CXCR4 on malignant acute leukemia cells is associated with extramedullary organ infiltration (149). *In-vivo* imaging studies of fluorescently labelled leukemic cells identified that homing of these cells to the bone marrow is dependent on the interaction of SDF-1 and its receptor CXCR4 (150). AMD3100 blocks CXCL12 binding and signaling through CXCR4 and is in phase I clinical trial for ALL (**Table 2**).

**Table 2:** Clinical trials investigating therapies for hematological malignancies based on targets involved in inflammation.

Therapeutic agent	Target	Tumor type	Clinicaltrials.gov ID	Phase
Plerixafor (AMD3100)	Blocks CXCL-12 binding to and signaling through CXCR4	R/R AML, R/R ALL, secondary AML/MDS, AML, ALL	NCT01319864	Phase I
BL-8040 in Combination with Nelarabine	Targeting CXCR4 signaling	R/R T-ALL/LBL	NCT02763384	Phase I
Ruxolitinib	JAK1/JAK2 inhibitor	R/R ETP-ALL in combination with chemotherapy	NCT03613428	Phase I/II
Buparlisib (BMK120)	PI3Ki	R/R acute leukemia Hematological malignancies	NCT01396499 NCT01833169	Phase I Phase II
Ibrutinib in combination with venetoclax and obinutuzumab	BTK signaling	R/R CLL	NCT03701282 NCT03737981	Phase III Phase III
Ibrutinib in combination with fludarabine and umbralisib	BTK Signaling	R/R CLL mantle cell lymphoma	NCT02268 NCT02514083	Phase II Phase II
Ibrutinib in combination with pembrolizumab and fludarabine	BTK Signaling	R/R CLL	NCT03204188	Phase II
Brontictuzumab (OMP-52M51)	Targets NOTCH-1	R/R lymphoid malignancies	NCT01703572	Phase I
LY3039478	Oral notch signaling inhibitor	T-ALL/T-LBL in combination with dexamethasone	NCT02518113	Phase I/II
Everolimus (rapamycin, RAD001)	mTOR inhibitor	pediatric ALL with chemotherapy	NCT01523977	Phase I
Temsirolimus (CCI-799)	mTOR inhibitor	relapsed ALL or NHL	NCT01403415	Phase I
Venetoclax (ABT-199)	targets BCL2	Naive AML with chemotherapy	NCT02203773	Phase Ib
		R/R ALL with chemotherapy	NCT03808610	Phase I/II
Glasdegib (PF-04449913)	oral inhibitor of the hedgehog pathway	AML or high risk MDS with chemotherapy	NCT01546038	Phase Ib/II
Quizartinib	oral FLT3-inhibitor	R/R AML	NCT02039726	Phase III
AG-120 (Ivosidenib) AG-221 (Enasidenib)	IDH1 inhibitor IDH2 inhibitor	R/R AML or R/R MDS FDA approved for AML R/R AML with an IDH2 mutation.	NCT02074839 NCT01915498	Phase I Phase I/II
Idelalisib (CAL-101)	PI3Kδ inhibitor	R/R ALL	NCT03742323	Phase I/II
Dactolisib (NVP-BEZ235)	Dual PI3K/mTOR inhibitors	R/R acute leukemia	NCT01756118	Phase I

Mutation in IL7R on pre-B-cells is known to cause B-cell oncogenesis. In addition, several shreds of evidence indicate that the IL-7/IL-7R axis may promote lymphoid-related leukemogenesis and modulate leukemic cell responses to some antineoplastic therapies (151–154).

The ETV6-RUNX1 (TEL-AML1) fusion gene that results from t (12, 21) (p12; q21) translocation is the most frequent genetic aberration reported in childhood ALL and known to have a putative prenatal first lesion (155, 156). ETV6-RUNX1 fusion protein binds to a principal TGF-β signaling target, Smad3, and blocks the ability of TGF-β to suppress the proliferation of pre-pro-B cells, which leads to leukemogenesis (157). Activation of STAT3 in ETV6-RUNX1 positive ALL via RAC1 is responsible for the survival, proliferation, and self-renewal of leukemic cells by upregulating MYC gene (158).

Translocations in the mixed lineage leukemia (MLL) gene account for >50 fusions that may participate in transforming BM cells through the regulation of *HOX* genes. MLL translocations are predominantly seen in infant B-ALL (<1 year of age) and 15% of adult ALL patients (159–161). LAMP5 (a member of the lysosome-associated membrane protein (LAMP) family) is known to regulate type 1 interferon (IFN-1) and pro-inflammatory signaling downstream of TLR9 activation (162). In mixed lineage leukemia-rearranged (MLL-r) leukemia, downregulation of LAMP5 led to inhibition of NFκB signaling and increased activation of type-1 interferon signaling downstream of Toll-like receptor/interleukin 1 receptor activation *in-vivo* and *in-vitro* (163).

The expression of the BCR/ ABL1 fusion gene due to translocation t (9, 22) (q34; q11) causes 5% of pediatric and 25% of adult ALL cases (164). STAT5, the regulator of immune function is responsible for leukemic cell proliferation and survival, and its deletion results in cell cycle arrest followed by apoptosis of BCR-ABL1 positive malignant B cells (165). Patients with JAK1/2 mutations and patients with the BCR-ABL1 fusion have both been found to share similar gene expression profiles and are associated with a poor prognosis (166). IL-7R causes activation of STAT5 by activating JAK1 and JAK2; mutation in IL-Rα identified in 2-3% of B-ALL cases cause constitutive activation of JAK-STAT signaling. In addition, all B-ALL cases with JAK2 mutations overexpress CRLF2 (type I cytokine receptor subunit, also known as thymic stromal lymphopoietin receptor) (167, 168). CRLF2 directly interacts with the tyrosine kinase JAK2 and helps promote the proliferation of normal and leukemic B cells. E2A-PBX1, another fusion protein commonly seen in B-ALL expression of the WNT-16 gene, which ultimately promotes the aberrant proliferation and survival of B-lineage cells (169).

### T-ALL

T-cell acute lymphoblastic leukemia is an aggressive blood cancer that comprises 10–15% of pediatric and ~25% of adult ALL cases, develops from the neoplastic transformation of T-cell precursors and their infiltration into BM and peripheral blood (PB) (170, 171). Aberrant Notch1 signaling plays a pivotal role in T-ALL leukemogenesis (170, 171). CNS infiltration risk is high in T-ALL patients and contributes to poor prognosis. A study on T-ALL patients and cell lines revealed that oncogenic Notch-1-induced chemokine CCR7 expression induced CNS infiltration and directional metastasis (172).

The role of IL-7 in the expansion and acceleration of leukemia progression has been revealed by engrafting T-ALL cell lines and primary T-ALL samples in immunocompromised mouse models after IL7 KO. It was shown that the IL-7/IL-7R axis causes activation of the PI3K/PKB/AKT signaling pathway resulting in downregulation of p27kip1 CDK inhibitors and upregulation of Bcl-2, promoting cell cycle progression and viability of T-ALL cells (173–175). Venetoclax (ABT-199) that targets higher BCL-2 expression is in phase-I clinical trials as monotherapy for R/R malignancies including T-ALL (NCT03236857) and in combination with Low-Intensity Chemotherapy and Venetoclax in phase I/II for R/R B or T-ALL (NCT03808610). Oligonucleotide microarray technology and pathway analysis in a study confirmed the pivotal role of IL-7 and CXCL12 in B and T-ALL (176). Activating mutations in the interleukin 7 receptor alpha chain (IL7R), Janus kinases, JAK1 or JAK3, or the Signal transducer and activator of transcription 5B (STAT5B) cause constitutive activation of JAK-STAT signaling observed in one-third of T-ALL patients (177–179).There are several ongoing clinical trials targeting the JAK/STAT pathway in T cell malignancies, which include NCT03613428, a phase I/II study combining ruxolitinib with the combination of vincristine, prednisone, and asparaginase in relapsed and refractory T-ALL (180). In T-ALL, soluble r-TRAIL failed to mediate apoptosis due to its low surface expression of death receptors DR4/DR5 in primary samples and cell lines (181).

PTEN-deficiency together with NRTK2 overexpression in T-ALL, caused activation of JAK/STAT3 and PI3K pathways, leading to aggressive disease, poor prognosis, and chemoresistance. The combined inhibition of phosphoinositide 3-kinase and STAT3 significantly suppressed the proliferation of PTEN-mutant T-ALL in culture and mouse xenografts (182). Also, array comparative genomic hybridization and sequence analysis from 44 pediatric DNA samples confirmed mutations in PI3K, PTEN or AKT ~48% T-ALL cases (183). PI3K, mTOR and PI3K/mTOR dual inhibitors including Buparlisib, Temsirolimus and Dactolisib are in clinical trials (**Table 2**).

CXCL12-binding receptor, commonly known as CXCR7 (CXC chemokine receptor 7), is highly expressed in T-ALL patient samples and cell lines and is responsible for T-ALL cell migration in response to CXCL12 induction (184). HTLV1-Tax (human T-cell leukemia virus, type-1 induced Tax) protein mediates HTLV1 viral-induced tumorigenesis in T-ALL by activating NFκB signaling (185). In addition, it was found that in tax-transformed cell line PX-1 is a T-ALL cell line which is transformed by the HTLV1-Tax protein. Inhibition of RelA (NFκB p65) using anti-sense oligonucleotides retarded the tumor growth of PX1 xenografts, suggest the importance of NFκB in HTLV1 associated tumors (186).

### CLL

B-cell chronic lymphocytic leukemia (CLL) is characterized by aberrant accretion of mature clonal CD5+ B lymphocytes in the blood, bone marrow and lymphoid tissues. These differentiated B cells display characteristic immunophenotypes expressing CD23, CD19 and low surface membrane immunoglobulin levels. CLL is of two subtypes: unmutated-CLL which arises from a naive B cell that has encountered antigen but with insufficient stimulus to form a germinal center (GC), and *IGHV* mutated-CLL (M–CLL) which arises from a memory cell that, following antigen encounter, has undergone somatic hypermutation (187).

Pro-inflammatory cytokines and chemokines like IFN-γ, interleukin 6 (IL-6), IL-10, IL-8, and TNF-α are found to be significantly high in untreated CLL patients (188–190). IL-4 receptor levels are constitutively high in CLL cells (191), which stimulates the JAK/STAT pathway that protects CLL cells from chemotherapy-induced apoptosis (192). The serum of CLL patients was found to have high amounts of TNF superfamily member BAFF (B-cell activation factor of the TNF family). It is known to rescue B-CLL cells from apoptosis (193). In normal B-Cells, the binding of antigen causes signalosome activation by kinases that lead to the regulated activation of downstream NFκB, PI3K/AKT and MAP kinase pathways, which are necessary for B-cell proliferation and survival (194). In contrast, in CLL, stimulation of the BCR induces expansion of the malignant clone (187, 195). In CLL, NFκB is constitutively stimulated by various extrinsic and intrinsic stimuli, and NFκB is the critical regulator for survival and differentiation in B-cells. Antigens from the microenvironment and intra-BCR self-antigens trigger BCR signaling, leading to the recruitment of tyrosine kinases that phosphorylate the immunoreceptor tyrosine-based activation motifs (ITAMs) of Ig-α/Ig-β (196). This induces activation of Bruton's tyrosine kinase (BTK), phosphoinositide 3- kinase (PI3K), and Ras- dependent extracellular signal-regulated kinase (ERK) (197), which ultimately leads to the upregulation of NFκB which promotes CLL-B cell survival (198).

BCR signaling in CLL is heterogeneous. CLL cells from some patients do not respond to antigen engagement when IgM is used for BCR stimulation, whereas cells from other patients retain their signaling capacity (199). Unlike normal B cells that undergo apoptosis, unless they differentiate into plasma or memory cells, CLL cells represent constitutive BCR activation, which causes activation of NFκB and NFκB-regulated genes (200), induction of pro-survival signals, and production of pro-inflammatory cytokines.

CLL cells require stimulus from the microenvironment for their survival. Macrophage migration inhibitory factor (MIF), a pro-inflammatory cytokine, is overexpressed and supports tumor growth in CLL patients (201) by stimulating signaling pathways, such as MAPK, NFκB, and AKT, on binding to receptors CD74 and CXCR2/CXCR4 (202–204). In B-cells, activation of the AKT and NFκB pathways via MIF leads to the production of IL-8, leading to the up-regulation of BCL-2, which provides apoptotic resistance to blasts (205, 206). CLL disease is known for its clinical and prognostic heterogeneity, which is found to be associated with BCR encoding genes and RNA binding protein-zeta-associated protein of 70 kDa (ZAP70) (187, 195). Patients with BCR encoded by unmutated immunoglobulin variable heavy-chain genes (*IGHV*) (206, 207) along with ZAP70 expression (207–209) represent aggressive disease phenotype as compared to normal B cells or most CLL cases with mutated IgVH that lacks ZAP70 expression. ZAP-70 induction in CLL B cells causes activation of specific BCR-signaling molecules, including SYK, BLNK, ERK, JNK, PLCγ, and AKT kinases (210, 211), indicating ZAP-70 promotes the growth and survival of the tumor cells by stimulating BCR signaling. It is found that ZAP70 contributes to the more aggressive clinical behavior in CLL by enhancing BCR-mediated signaling through the NFκB pathway (212).

There are many drugs which target tyrosine kinases like Ibrutinib, acalabrutinib and Zanubrutinib which irreversibly inhibit Bruton’s Tyrosine kinase (BTK) by binding to the cysteine residue in its active site. BTK is a kinase that is involved in multiple signaling pathways and plays a role in B-cell and myeloid cell progression and survival and therefore becomes a therapeutic target for hematological malignancies. Ibrutinib was approved in a randomized clinical trial CLL patients including R/R CLL patients. In the phase 3 trials ibrutinib was administered in combination with obinutuzumab (anti-CD20 monoclonal antibody) which increased overall survival rate of CLL patients (213–215). Many clinical trials have taken place in using a triple combination (Ibrutinib, obinutuzumab and venetoclax) to treat high-risk CLL and R/R CLL (216–218). The cells from ibrutinib treated CLL patients showed increased expansion of CD-19 targeted CAR-T Cells (CTL019) and had also reduced the expression of PD-1 on T cells and CD200 on B cells (219). Randomized clinical trials are also taking place in CLL and SLL patients to check the efficacy of CAR-T cells targeting CD19 (autologous CART-19 cells) (NCT01747486).

Acalabrutinib has also been used in the treatment of CLL and SLL patients. It was approved as a monotherapy in 2019 even after limited efficacy (220). Phase III clinical trials are undergoing with triple combination (Acalabrutinib, obinutuzumab and venetoclax) in CLL and SLL patients (NCT03836261). It has been reported that long -term administration of acalabrutinib leads to ventricular arrhythmias and sudden deaths whereas the combined therapy decreases these incidences (221).

## Role of RNA-binding proteins in inflammation

The effective activation and resolution of immune responses rely on the production and posttranscriptional regulation of mRNAs encoding inflammatory effector proteins. The association of RNA-binding proteins (RBPs) with mRNAs is essential in regulating their splicing, maturation, stability, and translation. In addition, several RBPs are reported to have a role in the modulation of the inflammatory response by controlling the expression of these inflammatory mRNAs and their decay.

**Figure 4:**
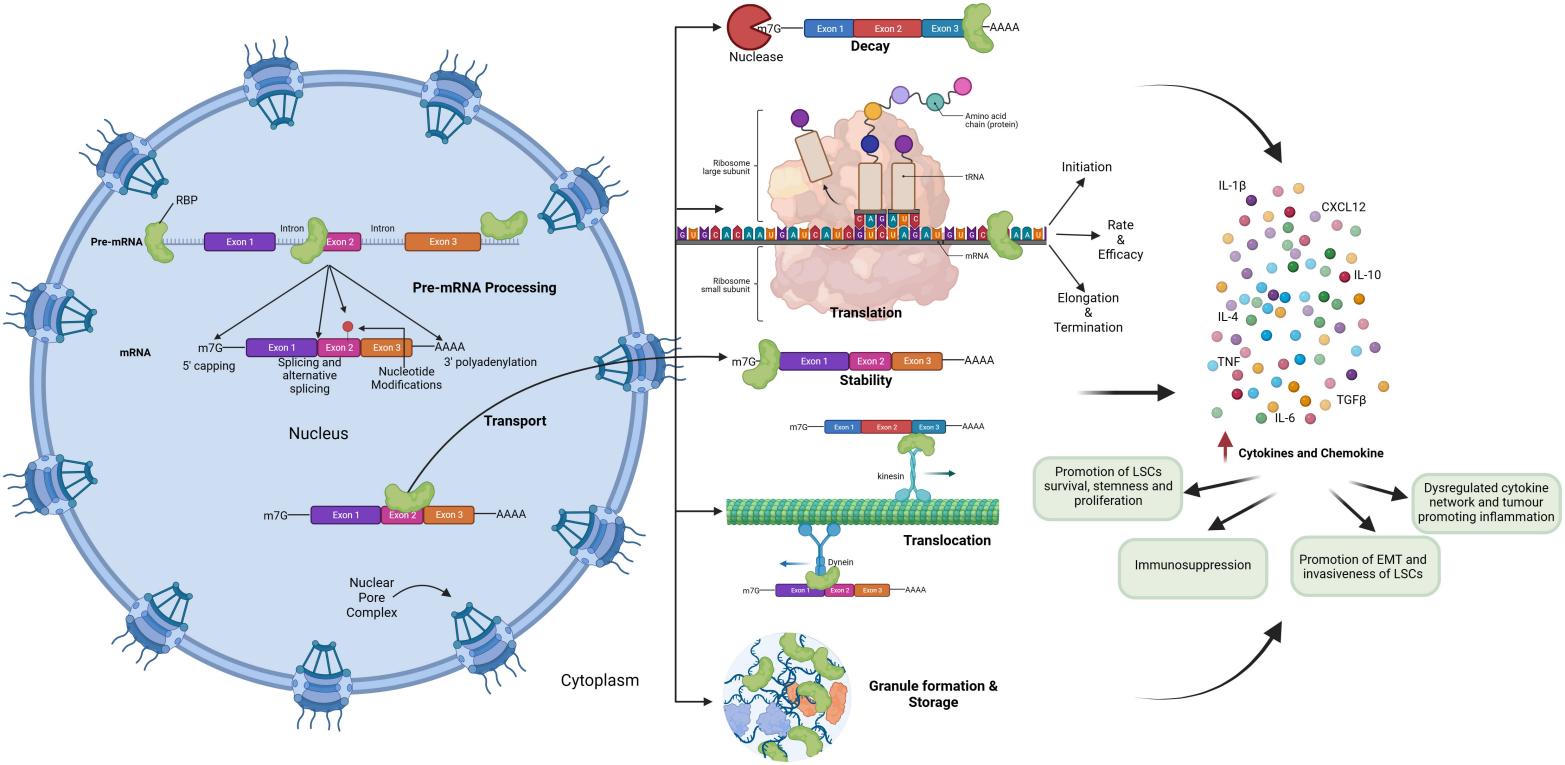
Role of RNA Binding Proteins in hematological malignancies by regulating inflammatory signaling.

RBPs mediate the regulation of inflammatory cytokine mRNAs like TNF-α, IL-10, and IL-6 by binding to their AU-rich elements near 3’-UTR and regulating mRNA stability, translation, and mRNA decay, thereby playing a role in inflammation-induced cancer development and progression (**Figure 4**).

### ZFP36

Cytokine mRNAs such as TNF-α have shorter half-lives and undergo decay through RBPs mediated regulation of AU-rich elements (ARE) (222). The ZFP36 family of RBPs consists of three family members namely TTP, ZFNB6L1 and ZFNB6L2.

Tristetraprolin (TTP) encoded by the ZFP36 gene is a well-characterized member of the TTP family of RBPs, with its role specifically involved in the regulation of the immune system. These RBPs bind to the ARE in the 3’-UTR of target mRNAs and regulate the mRNA half-lives and decay (223, 224). The ZFP36 family plays a significant role in attenuating inflammatory responses by inhibiting the production of cytokines such as TNF-α, IL-6 and IL-10 in macrophages (225).

TTP is considered a major mRNA destabilizing protein involved in the regulation of immune cells. TTP inhibits mRNA translation and mediates decay by recruitment of mRNA destabilizing proteins such as CCR4-NOT deadenylation and decapping complexes to the TTP-bound target mRNAs. TTP functions to resolve inflammation by controlling the mRNA translation and decay of pro-inflammatory cytokines mRNAs such as TNF-α and NFκB- pathway-related signaling molecules like TNF, CSCL2, and CXCL3. Conversely, it also induces inflammation by binding to mRNAs of inflammatory inhibitors like IER3 and DUSP1, thereby mediating accurate regulation of LPS-induced inflammatory response and resolution of inflammation (226, 227).

ZFNB6L1 and ZFNB6L2 proteins act similarly to TTP and attenuate the expression of their target mRNAs. ZFP36L1 and ZFP36L2 play a crucial role in the development of B-lymphocytes by regulating quiescence. Quiescence is crucial for facilitating variable-diversity-joining (VDJ) recombination in developing B-cells and the ZFP36L1 and ZFP36L2 proteins are responsible for maintaining quiescence before the expression of the precursor B cell receptor (pre-BCR). ZFP36L1 and ZFP36L2 also restore quiescence following pre-BCR induced expansion by suppressing the expression of mRNAs such as *Cyclin D3*, *Cyclin E2* (228, 229).

ZFNB6L1 and ZFNB6L2 regulate T-cell development by inhibiting the expression of Notch1. Double knockout of *ZFNB6L1* and *ZFNB6L2* in mice led to an abnormal increase in the NOTCH signaling pathway in double negative thymocytes and led to the development of T-ALL (230).

TTP primarily functions as a tumor suppressor gene in MYC-induced tumors. In a Myc-induced lymphoma model, TTP was found to be downregulated and restoration of TTP in these tumors led to the decay of mRNAs of *Fst1*, a pro-inflammatory cytokine and CCND1 thereby impairing the development and maintenance of lymphomas (231).

### HuR

Human Antigen R, also known as ELAVL1, is one of the widely studied RBPs involved in tumorigenesis. HuR and ZFP36 share many 3’-UTR ARE binding target mRNAs. HuR, like the ZFP36 family of RBPs, binds to ARE in 3’-UTR of target mRNAs.

In contrast to the ZFP36 family, HuR increases the stability of its target mRNAs (164). HuR binds to pro-inflammatory cytokine mRNAs like *COX-2*, *IL-2*, *IL-6*, *IL-8*, *IL-17*, *TNF-α*, *TGFβ* and *CXCL8* and increases their stability (232–236). HuR plays an indirect role in promoting Barrett’s esophagus associated carcinogenesis which is associated with chronic inflammation caused due to gastric acid reflux. HuR binds to iNOS mRNA through ARE elements at the 3’-UTR and stabilizes its mRNA thereby increasing the expression of iNOS. Inflammation induced over production of NO at the gastro-esophageal junction (GEJ) activates Caudal type homeobox (CDX2), a biomarker for Barrett’disease (237, 238).

Since HuR mainly functions to stabilize the target mRNAs, increased cytoplasmic expression of HuR has been found to be associated with various cancers such as oral, gastric, lung, breast, ovarian and renal cancers (239–244). Studies have shown that HuR expression clinically correlates with increased tumor size and higher tumor grade in breast cancer (245). Association between tumor stage and HuR expression was also seen in uterine cervical carcinoma along with non-small cell lung carcinoma (246, 247).

Apart from cancers, HuR also regulates B-cell and T-cell development in the immune system. Conditional knockout of HuR in mice models revealed that the population of pre-B-cells was reduced in bone marrow and follicular B-cells in the spleen and had significantly lower titers of serum immunoglobulins after knockout of HuR. HuR regulates splicing of mRNAs such as dihydrolipoamide S-succinyl transferase (DLST), a subunit of the 2-oxoglutarate dehydrogenase (α-KGDH) complex. Deletion of HuR led to disruption of mitochondrial metabolism and production of increased level of reactive oxygen species attributing to B-cell death (248). Thymocyte specific deletion of HuR in mice models showed that HuR is critical for T-cell development. Mice with deletion of HuR led to enlargement of thymus and loss of peripheral T- cells leading to lymphopenia (249).

HuR also has a role of polarizing macrophages to the M1 phenotype in the presence of LPS which is a systemic inflammatory stimulus. Interestingly, in a mouse model of LPS induced colitis and colorectal cancer, activated inflammatory tumor-activated macrophages from HuR-deficient mice showed increased expression of RNAs like *TNF*, *TGF-β*, *IL10*, *Ccr2* and *Ccl2*. Overexpression of HuR in myeloid cells induced posttranscriptional silencing of these inflammatory cytokine mRNAs, thereby protecting mice from colon cancer development (177, 178). This demonstrates a heterogenous response of HuR to bound mRNA targets which may be tissue specific translational silencing Another myeloid specific HuR overexpression model also demonstrated a downregulation of TIA-1 and cytokines such as TNF, IL-1β, and TGFβ1 (250).

### RNA-binding motif protein 39

This gene is also known as CAPER/RNPC2. It plays an important role in pre-mRNA splicing and regulates steroid hormone receptor mediated transcription (251). Its higher expression is associated with several malignancies such as TNBC, non-small cell lung cancer, colorectal adenocarcinomas and AML (252). RBM39 also acts as the activator of NFκB through its interaction with transcriptional activation domain of v-rel protein. Deletion of RBM39 has been found to suppress the oncogenic activity of NFκB in lymphocytes (253), proliferation of breast cancer cells and abrogates phosphorylation of c-Jun (254). Its role has been established in multiple myeloma along the HIF1α/DARS-AS1/RBM39 axis that could be a useful target in multiple myeloma (255).

### IGF2BPs

Insulin-like growth factor binding protein (IGF2BPs) are oncofetal proteins seen to be upregulated in various cancers, including different subtypes of B-ALL (256, 257). The IGF2BP family consists of three proteins that share sequence and functional homology, namely IGF2BP1, IGF2BP2, and IGF2BP3. These proteins are overexpressed during embryonic development, and re-expression is seen during the malignant transformation of cells. Overexpression of IGF2BP1 is seen in multiple epithelial tumors such as breast, pancreatic, and colon cancers (256). IGF2BP3 overexpression is also linked to numerous cervical, hepatocellular, breast and glial tumors (258). IGF2BP3 is overexpressed in the MLL translocated subtype of B- ALL, and IGF2BP1 is seen to be overexpressed in the ETV6-RUNX1 subtype of B-ALL (259, 260). This RBP family is known to influence the cytoplasmic fate of target mRNAs by regulating the translation, stabilization, location, and decay. IGF2BPs are also known to recruit mRNA stabilizers like ELAVL-1 (HuR) proteins (261).

IGF2BPs overexpression is well demonstrated in various epithelial cancers and leukemia, but the role of IGF2BPs in the induction of immune response has been recently elucidated.

One of the mechanisms of IGF2BPs mediated tumor progression is the regulation of tumor-associated inflammation. IGF2BP3 was found to bind and stabilize genes involved in the pro-inflammatory JAK/STAT signaling pathway and the ErbB signaling pathways in RS4;11 and Reh (B-ALL) cell lines. Overexpression of IGF2BP3 in the mouse bone marrow led to an expansion of progenitors belonging to all lineages (258, 262).

Similarly, IGF2BP1 targets were also identified in Reh cell line using RIP-seq and RNA-seq after IGF2BP1 knockout. The TNF-α induced NFκB pathway was one of the top targets of IGF2BP1 which was also reflected in the ETV6-RUNX1 positive B-ALL tumors (263). IGF2BP1 overexpression led to the stabilization of ubiquitin ligase receptor β*-TrCP1* mRNA, which in turn caused activation of the NFκB pathway through enhanced degradation of IKBs (264). IGF2BP1 also regulates the glial cells' inflammatory responses by stabilizing the target mRNAs *Gbp11 and Cp* (265).

However, in mouse models of melanoma, it was observed that knockdown of IGF2BP1-3 led to an increase in the expression of pro-inflammatory interferon signaling genes like IFI44 and OAS1 reiterating the fact that RBP modulation of target mRNA half-lives is tissue or context specific (264). Similarly, in a colon cancer mouse model, it was observed that stromal expression of IGF2BP1 was critical for the inhibition of the growth of colon cancer. IGF2BP1 KO led to a global increase in pro-inflammatory cytokines and chemokines like IL-6, IL1β and MCP1 (261).

Mechanistically, target mRNA stabilization of IGF2BPs has been revealed to be dependent on an N-6-methyladenosine (m6A) RNA modification on mRNAs- IGF2BPs function as readers of m6A RNA modification present near 3’-UTRs and promote mRNA stability and translation of target mRNAs (266). IGF2BP2 functions as a regulator of macrophage phenotype. IGF2BP2 mediates the switch from M1 to M2 phenotype by binding to TSC1 and PPARα directly to regulate their expression in an m6A-dependent manner (267).

## Small molecule inhibitors designed for targeting RNA binding proteins

RBPs are essential for controlling post-transcriptional gene expression, which is involved in multiple aspects of RNA metabolism. RBPs play a significant role in enhancing the translation and stability of mRNAs, which in turn contributes to the development and spread of cancer. Multiple studies have revealed crucial small compounds to specifically target the interactions between RBPs and RNA. These inhibitors mainly target the RNA Binding domains of RBPs which are crucial for RBP-RNA interactions such as the RNA-recognition motif (RRM), hnRNP K homology (KH), and the zinc-finger domain (268–270). These molecules serve as valuable tools for the development of innovative therapies aimed at inhibiting the function of RBPs. The key inhibitors developed for targeting RBPs demonstrating the anticancer activity and potent promising therapeutics are summarized in **Table 3**.

**Table 3:** RBPs and their role in inflammation and hematological malignancies.

RBP	Function	Role in Inflammation	Role in Hematological Malignancies	Clinical Implications	Drugs/Inhibitors	References
ZFP36 (TTP)	mRNA destabilization	Inhibits cytokine production (TNF-α, IL-6)	Implicated in inflammation-induced cancer development and progression	Potential tumor suppressor; Restoration may impair lymphoma development	None specified	(271, 272)
HuR (ELAVL1)	mRNA stabilization	Increases stability of pro-inflammatory cytokine mRNAs	Associated with various cancers (e.g., breast, lung, ovarian)	Cytoplasmic expression correlates with tumor size and grade; Potential therapeutic target	HuR inhibitors (e.g., MS-444, H1N, Mitoxantrone, CMLD-2, Quercetin, dihydrotanshinone-I)	(223, 273)
IGF2BPs (1–3)	Regulate mRNA stability, translation, decay	Stabilize pro-inflammatory genes in JAK/STAT, ErbB pathways	Implicated in multiple cancers (e.g., B-ALL, breast, colon)	Overexpression linked to tumor-associated inflammation; Potential therapeutic targets	BTYNB, C_20_H_18_BrN_5_OS, Compound 7773, JX5, CWI 1-2	(274, 275)
RBM39 (CAPER)	Pre-mRNA splicing; NFκB activation	Regulates steroid hormone receptor-mediated transcription	Associated with multiple malignancies (e.g., TNBC, AML)	Higher expression in various cancers; Implication in NFκB activation	E7820	(127, 276, 277)

### Human antigen R

MS-444 was characterized as a small molecule by a competitive binding assay involving HuR and the ARE-RNA complex. This assay demonstrated that MS-444 inhibits the interaction between HuR and the AU rich element of the target mRNA (278). This inhibitor has been extensively studied as a potent inhibitor for *in vitro* and *in vivo* research linked to cancer, specifically in melanoma, glioma, and pancreatic carcinoma (279–281).

A novel inhibitor against HuR was discovered by confocal nano scanning screening approach, named H1N. It inhibits the adenosyl transferase activity at the 3'-terminal of the RRM3 motif in HuR, therefore blocking its contact with the target mRNA (282).

Studies have revealed that the bioactive flavonoid quercetin targets the binding of cytokine mRNAs such as TNFα and IL-6 identified through electrophoretic mobility shift assay (EMSA) (283, 284).

Another compound mitoxantrone was also screened and it prevented the formation of a stable complex between HuR and ARE of TNFα mRNA (285). Mesenchymal stem cells-based study revealed mitoxantrone also led to disruption of complex between HuR and SOX2 mRNA (286).

Using the same screening approach, DHTS (15, 16-dihydrotanshinone-I), another inhibitor of HuR was identified to target interaction with TNFα mRNA and exerted its effect in nanomolar range (287). Treatment of cells with DHTS exerted anti-cancer effects through decrease in cell growth and proliferation along with increase in cytotoxicity as seen in colon cancer cells and glioma cells (288, 289).

Fluorescence polarization assay was optimized for high throughput screening of identification of molecules that disrupts interaction of HuR to AREs of target mRNAs (290). This screening led to identification of six coumarin derivatives. CMLD-2 was the most potent HuR-ARE disruptor identified and showed anti tumor activity in breast, lung, colon and thyroid cancers (291–293).

Suramin an FD1-approved anti-trypanosomal drug, was shown to competitively bind to HuR to show anti-tumor effect in oral cancer cells (294). Trichostatin (TSA) and 5-Aza 2’deoxycytidine (AZA), known inhibitors of histone deacetylation and DNA methylation have shown to affect the nuclear-cytoplasmic translocation of HuR in order to modulate the estrogen receptor (ER) mRNA dependent on HuR leading to reduction of tumor burden in ER negative breast cancer cell lines (295) (**Table 3**).

## Future perspectives and discussion

Understanding the intricate interplay between inflammation, hematopoietic stem cells (HSCs), and the development of hematological malignancies is fundamental for advancing cancer immunology. This comprehension paves the way for targeted therapeutic interventions, emphasizing the importance of unravelling complexities within the tumor microenvironment.

Efforts to modulate inflammatory pathways, especially those involving NFκB and STAT3, hold promise for therapeutic advancements. Inhibiting these pathways may disrupt the pro-survival signals in cancer cells, potentially sensitizing them to conventional treatments. A strategy aimed at suppressing chronic inflammation, possibly through anti-inflammatory agents, could be explored to prevent the initiation and progression of hematological malignancies.

The advancements in precision medicine and genetic profiling make it possible to treat cancer in a more personalized manner. Identifying specific genetic alterations associated with the activation of inflammatory pathways in individual patients could lead to targeted therapies. For instance, patients with mutations in NFκB or STAT3 pathways might benefit from tailored interventions aimed at restoring normal signaling.

Immunotherapeutic strategies could be designed to harness the body’s immune system against leukemia stem cells (LSCs). Targeting surface markers such as CD44, potentially in combination with other treatments, may provide a selective approach to enhance the eradication of LSCs. Additionally, disrupting the leukemia stem cell niche, possibly through interference with CXCL12/CXCR4 signaling, could be explored to render LSCs more vulnerable to immune-mediated clearance. Understanding the dynamics of CD47-SIRPα interactions in the context of the tumor microenvironment may unveil novel strategies to overcome immunosuppression. The TRAIL pathway emerges as a potential target for inducing apoptosis in myeloid leukemia cells. Combining TRAIL-based therapies with other targeted agents, such as AKT inhibitors and p53 activators, may provide synergistic effects, leading to improved therapeutic outcomes.

Targeting the elevated pro-inflammatory cytokines (IL-1α, IL-1β, TNFα) in the microenvironment of B-ALL patients could also be explored as a therapeutic strategy. Modulating the cytokine milieu may disrupt the inflammatory support for leukemic cells and potentially enhance the efficacy of standard treatments.

Understanding the role of the tumor microenvironment in supporting malignant hematopoietic clones emphasizes the importance of modulating this niche. Innovative therapies could focus on remodeling the microenvironment to create an inhospitable terrain for LSCs while promoting the resurgence of normal HSCs. This might involve the manipulation of signaling molecules and cellular interactions within the niche.

The complexity of the interactions between inflammation, HSCs, and leukemia necessitates multidisciplinary collaboration. Integrating expertise from immunology, genetics, and oncology can facilitate a more holistic understanding of the disease mechanisms. Collaborative efforts could lead to the development of innovative treatment modalities that address both the malignant cells and their microenvironment.

The identification of specific RBPs, such as IGF2BPs, ZFP36 and HuR, as key players in modulating the inflammatory response present an opportunity for targeted therapeutic interventions. Developing drugs that selectively modulate the activity of these RBPs could offer a precise way to regulate the expression of inflammatory cytokines, potentially mitigating inflammation-associated cancer progression.

The role of insulin-like growth factor 2 binding proteins (IGF2BPs) in the regulation of tumor-associated inflammation has been elucidated in recent publications. Investigating the mechanistic aspects of how IGF2BPs influence the tumor microenvironment and immune response could reveal novel targets and methodologies for therapeutic intervention. Unravelling the specific pathways through which IGF2BPs modulate inflammation may offer new strategies for controlling cancer progression.

Investigating the potential crosstalk between RBPs and immune checkpoint molecules could provide insights into the regulation of immune responses in the tumor microenvironment. Understanding how RBPs influence the expression and function of immune checkpoint proteins, such as PD-1 and CTLA-4, may reveal additional layers of complexity in immune modulation within the context of cancer. Adopting systems biology approaches, including omics technologies, can help unravel the global impact of RBPs on the cancer transcriptome.

As research progresses, a deeper understanding of the heterogeneity in RBP expression across different cancers and individual patients may emerge. This knowledge could pave the way for personalized cancer therapies, tailoring treatment strategies based on the unique RBP profiles of patients, thus optimizing the efficacy of immunomodulatory interventions.

Translating these discoveries from the laboratory to clinical settings is crucial. Investigating the potential of RBPs as diagnostic or prognostic biomarkers could aid in stratifying patients based on their likelihood of developing inflammation-associated cancers.

## Author contributions

SS: Conceptualization, Writing – review & editing, Writing – original draft. IB: Writing – original draft, Writing – review & editing. RB: Writing – original draft, Writing – review & editing. MB: Writing – original draft, Writing – review & editing. JP: Writing – review & editing, Conceptualization, Funding acquisition.
